# Self-efficacy and breastfeeding outcomes in mothers of premature and term infants: a longitudinal study

**DOI:** 10.1590/2317-1782/20232022123en

**Published:** 2023-10-09

**Authors:** Débora Gabriela Fernandes Assunção, Maria Clara Lima da Cruz, Norrara Scarlytt de Oliveira Holanda, Ruth Batista Bezerra Fagundes, Ana Verônica Dantas de Carvalho, Ingrid Guerra Azevedo, Silvana Alves Pereira

**Affiliations:** 1 Maternidade Escola Januário Cicco - MEJC - Natal (RN), Brasil.; 2 Departamento de Fisioterapia, Universidade Federal do Rio Grande do Norte - UFRN - Natal (RN), Brasil.; 3 Faculdade de Ciências da Saúde do Trairí - FACISA, Universidade Federal do Rio Grande do Norte - UFRN - Santa Cruz (RN), Brasil.; 4 Universidade Católica de Temuco - La Araucanía, Temuco, Chile.

**Keywords:** Breast Feeding, Self Efficacy, Lactation, Premature, Newborn

## Abstract

**Purpose:**

To analyze the breastfeeding self-efficacy in mothers of premature and full-term newborns, in the period of 180 days, and to know the social and obstetric factors that influence the practice of maintaining the exclusive breastfeeding in the period of exclusive recommendation.

**Methods:**

Cohort with 44 mothers admitted to a public maternity hospital between January and October 2018. The mothers were divided into two groups: Premature and Full-term Groups. The Breastfeeding Self-efficacy Scale was applied in the immediate postpartum period, in addition to a questionnaire elaborated by the authors to collect sociodemographic and obstetric variables. The follow-up was done on the 30th, 120th and 180th days of the newborn’s life, by telephone. For the statistical analysis between the groups, the Statistical Package for the Social Sciences (SPSS) was used.

**Results:**

There was no difference in the self-efficacy score, nor in the obstetric and socioeconomic characteristics between the groups, except for family income (lower in the Premature Group - p = 0.031). The diet type was different on the 30th day after delivery (p = 0.023), with greater adherence to the exclusive breastfeeding in the Premature Group. No association was found between breastfeeding self-efficacy and exclusive breastfeeding practice in the 180-day period.

**Conclusion:**

In this sample, the breastfeeding self-efficacy was not related to the exclusive breastfeeding practice in the period of 180 days, in both groups. The premature group showed lower family income and greater adherence to exclusive breastfeeding on the 30th day postpartum.

## INTRODUCTION

Breastfeeding is considered the best form of feeding for newborns due to its nutritional value and because it acts on immune function, motor and cognitive development^(1,2)^. However, even though it is considered a simple technique and low financial cost, the rate of exclusive breastfeeding in Brazil still comprises a period far below the 180 days that are recommended by the World Health Organization^(1)^.

Studies show that biological^(2)^, psychological^(3)^, social^(4)^ and cultural^(5)^ factors may have an influence on the process of early interruption of breastfeeding exclusive, as well as in its maintenance for longer^(6)^. Political organization, economic and environmental conditions also seem to influence this indicator^(7)^.

In the expectation of tracking risk factors for early weaning, some studies^(8,9)^ have investigated whether outcomes such as age^(9)^, schooling^(9)^, and income^(9)^ can influence personal expectations about self-efficacy, defined as the ability to successfully develop a given behavior^(10)^.

These studies^(8,9)^ use the Breastfeeding Self-efficacy Scale (BSES), a scale that was developed in Canada, in 1999^(11)^, translated and adapted to the Portuguese Language in 2010^(12)^. However, some of these research focus only on mothers of term newborns (NB)^(8)^ or assesses only the period of hospitalization or a short period close to hospital discharge compared to the 180 days recommended by the World Health Organization^(9)^.

Knowing that the practice of exclusive breastfeeding is contoured and influenced by many variables over time, and that appropriate nutrition from birth is a key component of lifelong health^(2)^, it is necessary to investigate the factors and outcomes related to maintaining breastfeeding for a longer period. Similarly, it becomes important to evaluate and compare this practice in groups of infants born at term and preterm, since there is evidence that exclusive breastfeeding rates (EBF) tend to be lower when the NB is preterm^(13)^.

Breastfeeding a premature newborn (PTNB) is undoubtedly a challenge, because, besides presenting muscular hypotonia, physiological and neurological immaturity, they remain awake for short periods^(14)^. But, despite desirable, little success in breastfeeding is observed among mothers of PTNB^(14,15)^. The process is permeated by difficulties, that occur both during hospitalization and in the maternal experience after her return home^(16)^ and seems to be somewhat better the higher the level of education and previous positive breastfeeding experience^(17)^.

Therefore, this study aims to analyze the breastfeeding self-efficacy in mothers of preterm and full-term newborns during the 180-day period, and to know the factors that influence the practice of exclusive breastfeeding in the period of exclusive recommendation.

## METHODS

### Study design and context

This is a cohort prospective study, carried out with 44 puerperae from a public maternity hospital, between the months of January and October 2018, approved by the Research Ethics Committee, under the terms of Resolution 466/12, of the National Research Ethics Commission (CONEP). The maternity hospital in this study is an institution qualified by the Ministry of Health as a Reference Unit in Tertiary Care for High-Risk Pregnancy and a Child-Friendly Hospital, which contains 128 beds and performs about 3,500 deliveries per year.

### Sample definition

The sample size calculation was determined (G* Power software, version 3.1.9.4) considering the mean (23 ±17.7) and high (107±82.3) self-efficacy score in breastfeeding of puerperae assessed up to the 60th day^(18)^, Cohen's effect size of 1.68, power of 0.95 and α error of 0.05. The calculated value of puerperae was 11, however, a possible loss of 15% was considered between the three evaluation intervals (30, 120 and 180 days) and the minimum value of expected puerperae for each of the groups was 17.

### Sample and data collection

Puerperae in the immediate postpartum period, who had a landline or cell phone, were at least 24 hours and at most 48 hours postpartum, over 18 years of age, with no history of anxiety and/or depression were eligible for the study. Puerperae who were mothers of NBs with some type of congenital malformation were excluded, puerperae who had postpartum complications, hearing, visual, cognitive impairment, and those with some contraindication to breastfeeding.

All mothers who met the inclusion criteria were verbally informed of the research objectives and invited to participate in the study, by signing an informed consent form at the postpartum moment while on the ward.

The selected puerperal women were divided into two groups: mothers of newborns with gestational age ≤ 36 weeks and 6 days (Premature Group - PG) and mothers of newborns with gestational age ≥ 37 weeks (Term Group - TG).

To collect socioeconomic data, a questionnaire designed by the authors was applied with the mother during hospitalization. The questionnaire addressed, in addition to socioeconomic questions, obstetric and neonatal questions, whose variables were: age, marital status, occupation, type of housing, education, family income, type of delivery, gestational age at birth, maternal contact after birth, number of prenatal visits, number of pregnancies, classification of gestational age at birth, guidance on breastfeeding in prenatal previous breastfeeding, time intending to exclusively breastfeed, sex of NB, birth weight, classification of birth weight, type of diet at the time of the interview, diet on the 30th day, diet on the 120th day, diet on the 180th day, number of children, score on the scale, and classification on the scale.

Measurement of self-efficacy was performed via the BSES scale in the immediate postpartum period. The scale consists of three dimensions - magnitude, generalizability, and strength - and is based on four sources of information - personal experience, vicarious or observational experience, verbal persuasion, and emotional and physiological state. For each rated item, the woman assigns a score ranging from 1 to 5 (1 - Strongly Disagree, 2- Disagree, 3 - Sometimes Agree, 4 - Agree, and 5 - Strongly Agree). Self-efficacy was identified from the total sum of responses and classified as low when the score was between 33 and 118, medium between 119 and 137, and high between 138 and 165 points^(19)^. Subsequent to the summing, self-efficacy was considered as high - yes or no - with this “no” group being the summation among mothers who scored between 33 and 137.

**Figure 1:**
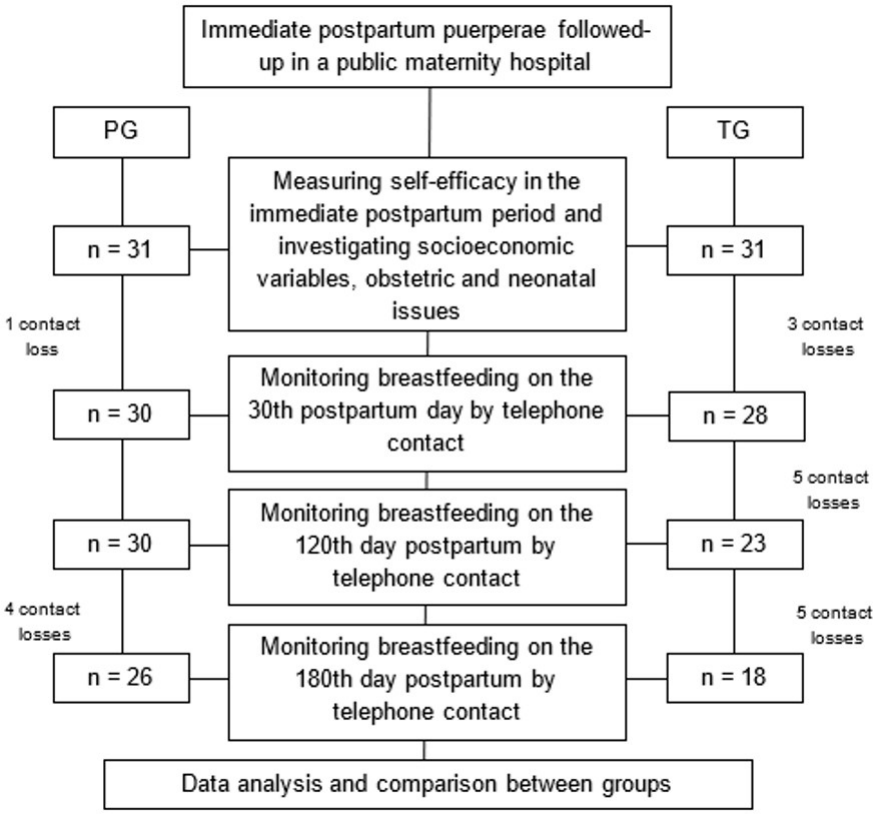
Graphical representation of the research steps *p-value of diet = 0.023, calculated by Fisher's Exact Test

Breastfeeding monitoring was monitored on the 30th, 120th and 180th days of the newborn's life, by telephone contact. In this stage, the researcher assessed the continuity of breastfeeding and classified it as exclusive or not. Exclusive breastfeeding (EBF) was defined according to World Health Organization criteria as no liquids or solids from any source other than that received from the breast^(20)^. On the telephone, mothers were asked about: “What kind of diet is the child getting?”, “Are you giving another milk or other food?”. The graphic representation of the research steps is detailed in Figure 1.

Caption: EBF = Exclusive breastfeeding

### Statistical analysis

The data were stored and analyzed in SPSS software (*Statistical Package for the Social Sciences*) version 20.0. The Kolmogorov-Smirnov test was used to evaluate the adherence of the variables to the normal distribution curve.

Pearson's Chi-square test was used to analyze socioeconomic characteristics, obstetric characteristics, breastfeeding rate at each moment of monitoring and between the groups (Premature and Term), and, when necessary, Fisher's Exact Test was used. The significance level adopted was p<0.05.

## RESULTS

### Socioeconomic, obstetric and neonatal characteristics

Among the 62 puerperae who started the study, 44 completed all stages (Figure 1) with a mean age of 26 years and a maximum age of 43 years, and 26 were allocated to the PG. Among the mothers who discontinued the research, 72% were from the TG, 78% were married or in a stable union, 72% had a family income of up to 1 minimum wage, and 67% had previous experience with breastfeeding.

The type of housing, marital status, occupation and education of the mothers were similar between the groups, while family income was lower in the SG (p=0.031), as shown in Table 1. In this group, 61% of the newborns were female, 57% were born by pelvic delivery and 73% of the mothers already had previous experience with breastfeeding. Table 2 shows the obstetric data of the sample.

**Table 1:** Socioeconomic characteristics of the Term Group and Premature Group

**Socioeconomic factors**	**Group**				**p-value**
	**Premature**		**Term**		
	**n (%)**		**n (%)**		
**Dwelling type**					
Own	11.0	(42.3)	9.0	(50.0)	0.614^a^
Rented/leased	15.0	(57.7)	9.0	(50.0)	
**Marital status**					
Married/stable union	23.0	(88.5)	12.0	(66.7)	0.128^b^
Single	3.0	(11.5)	6.0	(33.3)	
**Occupation**					
Salaried worker	11.0	(42.3)	7.0	(38.9)	0.340^a^
Unemployed	4.0	(15.4)	6.0	(33.3)	
Home Duties	11.0	(42.3)	5.0	(27.8)	
**Family income**					
1 minimum wage	21.0	(80.8)	9.0	(50.0)	0.031^a^*
2 or more minimum wages	5.0	(19.2)	9.0	(50.0)	
**Schooling**					
Incomplete elementary school	6.0	(23.1)	3.0	(16.7)	0.584^a^
Incomplete high school	8.0	(30.8)	4.0	(22.2)	
Complete high school	11.0	(42.3)	11.0	(61.1)	
Incomplete higher education	1.0	(3.8)	0.0	(0.0)	

a Pearson's Chi-square test;

b Fisher's Exact test;

* p<0.05

Caption: p = p-value; n = number of subjects included in each group

**Table 2:** Obstetric and neonatal characteristics of the Premature Group and Term Group

**Obstetric Characteristics**	**Group**				**p-value**
	**Premature**		**Term**		
	**n (%)**		**n (%)**		
**Gender of NB**					
Male	10	-38.5	10	-55.6	0.263^a^
Female	16	-61.5	8	-44.4	
**Type of delivery**					
Pelvic	15	-57.7	10	-55.6	0.888^a^
Caesarean	11	-42.3	8	-44.4	
**Birth weight classification**					
AGA	24	-58.5	17	-41.5	0.363^b^
LGA	1	-100	0	0	
SGA	2	-100	0	0	
**Prenatal breastfeeding guidance**					
Yes	16	-61.5	9	-55.6	0.691^a^
No	10	-38.5	8	-44.4	
**Previous experience with breastfeeding**					
Yes	19	-73.1	11	-61.1	0.402^a^
No	7	-26.9	7	-38.9	
**Number of total pregnancies**					
< 3	13	-50	10	-55.5	0.717^a^
≥ 3	13	-50	8	-45.5	

a Pearson's Chi-square test;

b Fisher's Exact Test

Caption: n = number of subjects included in each group; NB = newborn; AGA = Adequate for Gestational Age; LGA = Large for Gestational Age; SGA = Small for Gestational Age; p = p-value

Self-efficacy scores were similar between groups (p=0.354). For Table 3, self-efficacy was considered high or not high. No mothers were rated with low self-efficacy and 64% of the mothers reported high self-efficacy. For both groups, the declared self-efficacy was not related to EBF in any of the study steps (Table 3).

**Table 3:** Relationship of self-efficacy with exclusive breastfeeding on the 30th, 120th and 180th day after delivery

**Group**	**Follow-up time**	**EBF**	**High self-efficacy**		**p-value**
			**Yes**	**No**	
			**n (%)**	**n (%)**	
**Preterm**	**30th day**	**Yes**	18.0 (69.2)	8.0 (30.8)	-
		**No**	0.0 (0.0)	0.0 (0.0)	
	**120th day**	**Yes**	12.0 (66.6)	6.0 (33.4)	1.000
		**No**	6.0 (75.0)	2.0 (25.0)	
	**180th day**	**Yes**	6.0 (75.0)	2.0 (25.0)	1.000
		**No**	12.0 (66.6)	6.0 (33.4)	
**Term**	**30th day**	**Yes**	8.0 (57.2)	6.0 (42.8)	1.000
		**No**	2.0 (50.0)	2.0 (50.0)	
	**120th day**	**Yes**	8.0 (66.6)	4.0 (33.4)	0.321
		**No**	2.0 (33.4)	4.0 (66.6)	
	**180th day**	**Yes**	3.0 (75.0)	1.0 (25.0)	0.588
		**No**	7.0 (50.0)	7.0 (50.0)	

Caption: EBF = Exclusive breastfeeding; n = number of subjects included in each group; p = p-value PG = Premature Group; TG = Term Group; n = number of subjects included

**Figure 2:**
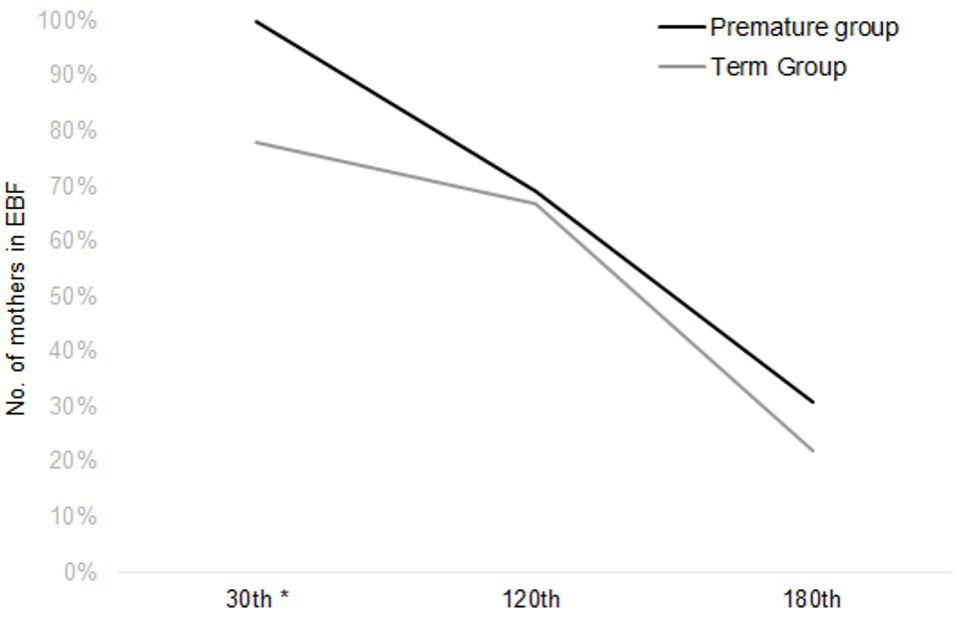
Number of mothers in EBF on the 30th, 120th and 180th day of follow-up

EBF compliance, for both groups, decreased progressively over the course of months. On postpartum day 30, the PG showed adherence of 100% of the mothers, versus 78% of the TG (p=0.023). Figure 2 presents these results.

## DISCUSSION

Although most mothers of preterm and full-term newborns had high expectations about breastfeeding self-efficacy, no association of self-efficacy and the practice of EBF within 180 days was found in the sample studied. In the analysis of socioeconomic characteristics, the group of mothers of premature newborns had lower family income when compared to the group of mothers of term newborns.

Monthly family income can cause changes in maternal infant health and influence birth conditions, as well as lead to neonatal and childhood morbidities^(21)^. Blencowe et al^(22)^ state that preterm birth rates are higher in low-income countries, followed by middle-income countries; which may account for the difference presented in our study.

Self-efficacy score did not differ between TG and PG and was not a predictor for EBF at any point in the research. Unlike this result, Brandão et al., in a prospective study^(23)^, conducted with mothers of newborns at term, in which they applied the BSES while still in the gestational period, found an association between the BSES classification and EBF on the 30th day, when they followed up the population until the 180th day of birth^(23)^. Not including mothers of preterm infants may have contributed to the success of the association in Brandão's study. Wang et al and Chantal et al^(15,24)^, highlight that PTNBs require greater attention, as they face physiological and neurodevelopmental obstacles related to their immaturity^(15)^. Depending on gestational age, PTNBs exhibit poor suction, difficulty with respiratory coordination and swallowing^(24)^.

However, in our study, although self-efficacy did not predict EBF adherence over the 180-day period, the PG showed greater EBF adherence across all assessments. A hypothesis to justify this result refers to the length of hospitalization, a variable not controlled in the present study. With longer hospital stay, compared to newborns at term, mothers of PTNBs have the opportunity to receive longer continuing education on breastfeeding and newborn care.

Corroborating the previously cited hypothesis regarding the length of stay of PTNBs in hospital environment, a study conducted in the immediate postpartum period, with education and support groups organized by health professionals specialized in the clinical management of breastfeeding and lactation, reported that educational interventions performed during hospitalization can help mothers to increase self-efficacy and maintain exclusive breastfeeding^(25)^.

Additionally, the length of hospital stay may have contributed to regular communication between families and health services, favoring engagement and adherence to the study after hospital discharge^(26)^. One data to justify this hypothesis of ours was the low adherence of the TG, a group of newborns that usually stays less time hospitalized, when compared to premature newborns^(27)^. Adherence to interventions by parents or guardians is also fundamental during newborn care, as it allows individualized goal setting and care optimization^(26)^.

Regarding the rate of EBF, on the 180th day, 27% of infants were exclusively breastfeeding; a result lower than that reported by the Ministry of Health in 2009^(1)^, in which the prevalence of EBF in children under 6 months was 41% in all Brazilian capitals and 40% in the city of Natal. Another study^(28)^, conducted in a child-friendly maternity hospital with 261 mothers, found that the rate of exclusive breastfeeding in the 6th month of life was 5.7%, a result lower than that found in this study.

In fact, early weaning is a complex phenomenon and different researchers try to understand which the best tool is to predict this risk. Cortelo et al^(29)^, in their study, used Antonovsky’s sense of coherence to explain this phenomenon, and the results pointed out that mothers with a greater sense of coherence are almost twice as likely to keep breastfeeding longer. Another study, in children younger than 1 year, related the Edinburgh Postpartum Depression Scale to breastfeeding in the last 24 hours before the scale was applied, the result found was a higher chance of absence of exclusive breastfeeding among mothers with symptoms of postpartum depression^(30)^.

It is known that the practice of EBF can be influenced by biological^(2)^, psychological^(3)^, social^(4)^ and cultural factors^(5)^. Although we investigated the practice of EBF over a long period, i.e., for 180 days, we were unable to track all risk factors that could interfere with this outcome. An example on this aspect was the sharp drop in EBF compliance from day 120 of the TG. We attribute this drop to the fact that many mothers return to their jobs after 120 days of leave, but we were unable to investigate this outcome. In addition, some variables that may interfere with the duration of breastfeeding, such as working outside the home and using pacifiers, were not analyzed.

We understand this difficulty to analyze some variables, which can be considered important, as a limitation of our study. Conducting a longitudinal study with mothers of newborns in Northeast Brazil is an arduous task, and we chose to keep the interview short and easy to conduct, in order to have time to empower the mothers about the importance of maintaining the EBF.

To have followed the puerperal women until the 180th day of life of NB was positive, since few studies can maintain such a long contact in this type of sample. However, the first time chosen for the application of the BSES scale may not have been the most reliable for reporting breastfeeding self-efficacy in mothers, given that it was performed in the immediate postpartum period (between the first 24 - 48 hours after delivery) and it is known that breastfeeding in the first hours of life is challenging, confusing and unfamiliar for both the mother (especially primiparous mothers) and the baby.

Therefore, it is suggested that further studies be conducted, with the evaluation done at times other than the immediate postpartum period, to confirm the findings. Another limitation is that the sample was conducted in only one maternity hospital. Therefore, further studies under this same perspective should be conducted in order to confirm the data evidenced in this population.

## CONCLUSION

In the sample studied, BSES does not constitute a predictive factor for early weaning in either mothers of preterm newborns or mothers of full-term newborns. The group of premature subjects showed higher adherence to exclusive breastfeeding on the 30th day postpartum, in addition to lower family income. Though, new studies should improve this monitoring system and investigate different factors that may present themselves as barriers to maintaining exclusive breastfeeding.
