# Supplementary material for: Self-efficacy and breastfeeding outcomes in mothers of premature and term infants: a longitudinal study
Source: Codas. 2023 Oct 9;35(5):e20220123. doi: 10.1590/2317-1782/20232022123en (PMC10688297; doi:10.1590/2317-1782/20232022123en)
Supplement: Translation

# Translation: Autoeficácia e desfechos da amamentação em mães de bebês prematuros e a termo: um estudo longitudinal

**DOI:** 10.1590/2317-1782/20232022123pt

**Authors:** Débora Gabriela Fernandes Assunção, Maria Clara Lima da Cruz, Norrara Scarlytt de Oliveira Holanda, Ruth Batista Bezerra Fagundes, Ana Verônica Dantas de Carvalho, Ingrid Guerra Azevedo, Silvana Alves Pereira

**Affiliations:** 1 Maternidade Escola Januário Cicco - MEJC - Natal (RN), Brasil.; 2 Departamento de Fisioterapia, Universidade Federal do Rio Grande do Norte - UFRN - Natal (RN), Brasil.; 3 Faculdade de Ciências da Saúde do Trairí - FACISA, Universidade Federal do Rio Grande do Norte - UFRN - Santa Cruz (RN), Brasil.; 4 Universidade Católica de Temuco - La Araucanía, Temuco, Chile.

**Keywords:** Aleitamento Materno, Autoeficácia, Lactação, Recém-nascido Prematuro, Recém-nascido

## Abstract

**Objetivo:**

Analisar a autoeficácia da amamentação, no período de 180 dias, em mães de recém-nascidos prematuros e a termo, e conhecer os fatores que influenciam na prática do aleitamento materno exclusivo no período de recomendação exclusiva.

**Método:**

Coorte realizado com 44 puérperas de uma maternidade pública, entre janeiro e outubro de 2018. As puérperas foram divididas em dois grupos: Grupo Prematuro e Grupo Termo. A *Breastfeeding Self-efficacy Scale* foi aplicada no pós-parto imediato, além de um questionário elaborado pelos autores para coleta de variáveis sociodemográficas e obstétricas. O monitoramento da continuidade do aleitamento materno foi feito no 30º, 120º e 180º dia de vida do neonato, via telefone. Para a análise estatística entre os grupos foi utilizado o *Statistical Package for the Social Sciences* (SPSS).

**Resultados:**

Não houve diferença na pontuação da autoeficácia, nas características obstétricas e socioeconômicas entre os grupos, exceto para a variável renda familiar, (menor no Grupo Prematuro - p=0,031). O tipo de dieta foi diferente no 30º dia pós parto (p=0,023), com maior adesão ao aleitamento materno exclusivo no Grupo Prematuro. Não foi encontrada associação entre a autoeficácia da amamentação e a prática do aleitamento materno exclusivo no período de 180 dias.

**Conclusão:**

Nesta amostra, a autoeficácia da amamentação não teve relação com a prática do aleitamento materno exclusivo no período de 180 dias em ambos os grupos. O grupo de prematuros apresentou menor renda familiar e maior adesão ao aleitamento materno exclusivo no 30º dia pós parto.

## INTRODUÇÃO

O aleitamento materno é considerado a melhor forma de alimentação para os recém-nascidos devido ao seu valor nutricional e por atuar na função imune, no desenvolvimento motor e cognitivo^(1,2)^. Entretanto, mesmo sendo considerada uma técnica simples e de baixo custo financeiro, a taxa de amamentação exclusiva no Brasil ainda compreende um período muito abaixo dos 180 dias que são recomendados pela Organização Mundial de Saúde^(1)^.

Estudos demonstram que fatores biológicos^(2)^, psicológicos^(3)^, sociais^(4)^ e culturais^(5)^ podem ter influência sobre o processo de interrupção precoce da amamentação exclusiva, como também na sua manutenção por mais tempo^(6)^. A organização política, as condições econômicas e ambientais também parecem influenciar esse indicador^(7)^.

Na expectativa de rastrear os fatores de risco para o desmame precoce, alguns estudos^(8,9)^ têm investigado se desfechos como idade^(9)^, escolaridade^(9)^, e renda^(9)^ podem influenciar as expectativas pessoais acerca da autoeficácia, definida como a capacidade de desenvolver com sucesso um determinado comportamento^(10)^.

Esses estudos^(8,9)^ utilizam a *Breastfeeding Self-efficacy Scale* (BSES), uma escala que foi desenvolvida no Canadá, em 1999^(11)^, traduzida e adaptada para a Língua Portuguesa em 2010^(12)^. No entanto, algumas dessas pesquisas se concentram apenas em mães de recém-nascidos (RN) a termo^(8)^, ou avaliam somente o período de internação ou um período curto próximo à alta hospitalar, se comparado aos 180 dias recomendados pela Organização Mundial de Saúde^(9)^.

Sabendo que a prática da amamentação exclusiva é contornada e influenciada por diversas variáveis ao longo do tempo, e que a nutrição apropriada desde o nascimento é um componente chave para a saúde ao longo da vida^(2)^, faz-se necessário investigar os fatores e desfechos relacionados à manutenção da amamentação por um período mais prolongado. Da mesma maneira, se torna importante avaliar e comparar essa prática em grupos de lactentes nascidos a termo e prematuros, uma vez que há evidências que as taxas de aleitamento materno exclusivo (AME) tendem a ser menores quando o RN é prematuro^(13)^.

Amamentar um recém-nascido prematuro (RNPT) é, sem dúvida, um desafio, pois, além de apresentarem hipotonia muscular, imaturidade fisiológica e neurológica, permanecem acordados por períodos curtos^(14)^. Mas, apesar de desejável, observa-se pouco sucesso na amamentação entre mães de RNPT^(14,15)^. O processo é permeado por dificuldades, que ocorrem tanto na internação quanto na vivência materna após seu retorno para o lar^(16)^ e parece ser um pouco melhor quanto maior o nível de escolaridade e experiência anterior de amamentação positiva^(17)^.

Diante disso, este estudo tem como objetivo analisar a autoeficácia da amamentação, no período de 180 dias, em mães de recém-nascidos prematuros e a termo, e conhecer os fatores que influenciam na prática do aleitamento materno exclusivo no período de recomendação exclusiva.

## MÉTODO

### Desenho do estudo e contexto

Trata-se de um estudo de coorte, prospectivo, realizado com 44 puérperas de uma maternidade pública, entre os meses de janeiro a outubro de 2018, aprovado pelo Comitê de Ética em Pesquisa, nos termos da Resolução 466/12, da Comissão Nacional de Ética em Pesquisa (CONEP). A maternidade deste estudo é uma instituição habilitada pelo Ministério da Saúde como Unidade de Referência em Atendimento Terciário à Gestação de Alto Risco e Hospital Amigo da Criança, que contém 128 leitos e realiza cerca de 3.500 partos por ano.

### Definição da amostragem

O cálculo do tamanho amostral foi determinado (G* Power software, version 3.1.9.4) considerando o escore de autoeficácia média (23 ±17.7) e alta (107±82.3) na amamentação de puérperas avaliadas até o 60º dia^(18)^, tamanho do efeito d de Cohen de 1.68, poder de 0,95 e erro α de 0,05. O valor calculado de puérperas foi de 11, entretanto, considerou-se uma possível perda de 15% entre os três intervalos de avaliação (30, 120 e 180 dias) e o valor mínimo de puérperas esperado para cada um dos grupos foi de 17.

### Amostra e coleta de dados

Foram elegíveis para o estudo puérperas no pós-parto imediato, que possuíam telefone fixo ou celular, tinham no mínimo 24 horas e no máximo 48 horas pós-parto, idade superior a 18 anos, sem histórico de ansiedade e/ou depressão. Foram excluídas as puérperas mães de RN com algum tipo de má-formação congênita, puérperas que apresentaram intercorrências no pós-parto, deficiência auditiva, visual, cognitiva, e aquelas com alguma contraindicação para amamentar.

Todas as mães que preenchiam os critérios de inclusão foram informadas verbalmente dos objetivos da pesquisa e convidadas a participar do estudo, assinando um termo de consentimento livre e esclarecido no momento pós-parto, enquanto estavam na enfermaria.

As puérperas selecionadas foram divididas em dois grupos: mães de recém-nascidos com idade gestacional ≤ 36 semanas e 6 dias (Grupo Prematuro - GP) e mães de recém-nascidos com idade gestacional ≥ 37 semanas (Grupo Termo - GT).

Para a coleta de dados socioeconômicos, foi aplicado com a mãe, durante a internação, um questionário elaborado pelos autores. O questionário abordou, além das questões socioeconômicas, questões obstétricas e neonatais, cujas variáveis eram: idade, estado civil, ocupação, tipo de moradia, escolaridade, renda familiar, tipo de parto, idade gestacional ao nascimento, contato materno após o nascimento, número de consultas no pré-natal, número de gestações, classificação da idade gestacional ao nascer, orientação sobre amamentação no pré-natal, amamentação anterior, tempo que pretende amamentar exclusivamente, sexo do RN, peso ao nascer, classificação do peso ao nascer, tipo de dieta no momento da entrevista, dieta no 30º dia, dieta no 120º dia, dieta no 180º dia, quantidade de filhos, pontuação na escala e classificação na escala.

A mensuração da autoeficácia foi realizada através da escala BSES no pós-parto imediato. A escala é constituída por três dimensões - magnitude, generalização e força - e está baseada em quatro fontes de informações - experiência pessoal, experiência vicária ou observacional, persuasão verbal e estado emocional e fisiológico. Para cada item avaliado, a mulher atribui uma pontuação variável de 1 a 5 (1 - Discordo totalmente, 2- Discordo, 3 - Às vezes concordo, 4 - Concordo e 5 - Concordo totalmente). A autoeficácia foi identificada a partir do somatório total das respostas e classificada em baixa quando a pontuação ficou entre 33 e 118, média entre 119 e 137 e alta entre 138 e 165 pontos^(19)^. Posterior ao somatório, a autoeficácia foi considerada como alta - sim ou não - sendo este grupo de “não” o somatório entre as mães que tiveram pontuação entre 33 e 137.

O monitoramento do aleitamento materno foi acompanhado nos 30º, 120º e 180º dias de vida do recém-nascido, por contato telefônico. Nesta fase, o pesquisador avaliou a continuidade da amamentação e a classificou em exclusiva ou não. O aleitamento materno exclusivo (AME) foi definido conforme os critérios da Organização Mundial da Saúde, como nenhum outro líquido ou sólido de qualquer outra fonte, além da recebida da mama^(20)^. Ao telefone, as mães foram questionadas sobre: “Qual tipo de dieta a criança está recebendo?”, “Está dando outro leite ou outro alimento?”. A representação gráfica das etapas da pesquisa encontra-se detalhada na Figura 1.

**Figura 1:** Representação gráfica das etapas da pesquisa. *p-valor da dieta = 0,023, calculado pelo Teste Exato de Fisher

Legenda: AME = Aleitamento Materno Exclusivo

### Análise estatística

Os dados foram armazenados e analisados no programa SPSS (*Statistical Package for the Social Sciences*) versão 20.0. O teste de Kolmogorov-smirnov foi empregado para avaliar a aderência das variáveis à curva de distribuição normal.

Para a análise das características socioeconômicas, obstétricas, taxa de aleitamento materno em cada momento do monitoramento e entre os grupos (Prematuro e Termo), foi utilizado o teste de Qui-Quadrado de Pearson, e, quando necessário, foi empregado o Teste Exato de Fisher. O nível de significância adotado foi p<0,05.

## RESULTADOS

### Características socioeconômicas, obstétricas e neonatais

Entre as 62 puérperas que iniciaram o estudo, 44 completaram todas as etapas (Figura 1), com média de idade de 26 anos e idade máxima de 43 anos, sendo 26 alocadas no GP. Dentre as mães que descontinuaram a pesquisa, 72% eram do GT, 78% eram casadas ou tinham união estável, 72% possuíam renda familiar de até 1 salário mínimo e 67% já tinham experiência anterior com amamentação.

O tipo de moradia, estado civil, ocupação e escolaridade das mães foi similar entre os grupos, enquanto a renda familiar apresentou-se menor no GP (p=0,031), conforme apresentado na Tabela 1. Neste grupo, 61% dos recém-nascidos eram do sexo feminino, 57% nasceram de parto pélvico e 73% das mães já tinham experiência anterior com amamentação. A Tabela 2 apresenta os dados obstétricos da amostra.

**Tabela 1:** Características socioeconômicas do Grupo Termo e Grupo Prematuro

**Fatores socioeconômicos**	**Grupo**				V**alor de p**
	**Prematuro**		**Termo**		
	**n (%)**		**n (%)**		
**Tipo de moradia**					
Própria	11,0	(42,3)	9,0	(50,0)	0,614^a^
Alugada/cedida	15,0	(57,7)	9,0	(50,0)	
**Estado civil**					
Casada/ União estável	23,0	(88,5)	12,0	(66,7)	0,128^b^
Solteira	3,0	(11,5)	6,0	(33,3)	
**Ocupação**					
Trabalhadora assalariada	11,0	(42,3)	7,0	(38,9)	0,340^a^
Desempregada	4,0	(15,4)	6,0	(33,3)	
Do lar	11,0	(42,3)	5,0	(27,8)	
**Renda familiar**					
Até 1 salário mínimo	21,0	(80,8)	9,0	(50,0)	0,031^a^*
2 ou mais salários mínimos	5,0	(19,2)	9,0	(50,0)	
**Escolaridade**					
Ensino fundamental incompleto	6,0	(23,1)	3,0	(16,7)	0,584^a^
Ensino médio incompleto	8,0	(30,8)	4,0	(22,2)	
Ensino médio completo	11,0	(42,3)	11,0	(61,1)	
Ensino superior incompleto	1,0	(3,8)	0,0	(0,0)	

a Teste Qui-quadrado de Pearson;

b Teste Exato de Fisher;

* p<0,05

Legenda: p = valor de p; n = número de sujeitos incluídos em cada grupo

**Tabela 2:** Características obstétricas e neonatais do Grupo Prematuro e Grupo Termo

**Características Obstétricas**	**Grupo**				**Valor de p**
	**Prematuro**		**Termo**		
	**n (%)**		**n (%)**		
**Sexo do RN**					
Masculino	10,0	(38,5)	10,0	(55,6)	0,263^a^
Feminino	16,0	(61,5)	8,0	(44,4)	
**Tipo de parto**					
Pélvico	15,0	(57,7)	10,0	(55,6)	0,888^a^
Cesariano	11,0	(42,3)	8,0	(44,4)	
**Classificação de peso ao nascer**					
AIG	24,0	(58,5)	17,0	(41,5)	0.363^b^
GIG	1,0	(100,0)	0,0	(0,0)	
PIG	2,0	(100,0)	0,0	(0,0)	
**Orientação sobre amamentação no pré-natal**					
Sim	16,0	(61,5)	9,0	(55,6)	0,691^a^
Não	10,0	(38,5)	8,0	(44,4)	
**Experiência anterior com amamentação**					
Sim	19,0	(73,1)	11,0	(61,1)	0,402^a^
Não	7,0	(26,9)	7,0	(38,9)	
**Número de gestações totais**					
<3	13,0	(50,0)	10,0	(55,5)	0,717^a^
≥3	13,0	(50,0)	8,0	(45,5)	

a Teste Qui-quadrado de Pearson;

b Teste Exato de Fisher

Legenda: n = número de sujeitos incluídos em cada grupo; RN = recém-nascido; AIG = Adequado para a idade gestacional; GIG = Grande para a idade gestacional; PIG = Pequeno para a idade gestacional; p = valor de p

A pontuação da autoeficácia foi similar entre os grupos (p=0,354). Para a Tabela 3, a autoeficácia foi considerada como alta ou não. Nenhuma mãe foi classificada com autoeficácia baixa e 64% das mães relataram autoeficácia alta. Para ambos os grupos, a autoeficácia declarada não apresentou relação com o AME em nenhuma das etapas do estudo (Tabela 3).

**Tabela 3:** Relação da autoeficácia com o aleitamento materno exclusivo no 30º, 120º e 180º dia pós parto

**Grupo**	**Tempo de acompanhamento**	**AME**	**Autoeficácia alta**		**Valor de p**
			**Sim n (%)**	**Não n (%)**	
**Prematuro**	**30º dia**	**Sim**	18,0 (69,2)	8,0 (30,8)	-
		**Não**	0,0 (0,0)	0,0 (0,0)	
	**120º dia**	**Sim**	12,0 (66,6)	6,0 (33,4)	1,000
		**Não**	6,0 (75,0)	2,0 (25,0)	
	**180º dia**	**Sim**	6,0 (75,0)	2,0 (25,0)	1,000
		**Não**	12,0 (66,6)	6,0 (33,4)	
**Termo**	**30º dia**	**Sim**	8,0 (57,2)	6,0 (42,8)	1,000
		**Não**	2,0 (50,0)	2,0 (50,0)	
	**120º dia**	**Sim**	8,0 (66,6)	4,0 (33,4)	0,321
		**Não**	2,0 (33,4)	4,0 (66,6)	
	**180º dia**	**Sim**	3,0 (75,0)	1,0 (25,0)	0,588
		**Não**	7,0 (50,0)	7,0 (50,0)	

Legenda: AME = Aleitamento materno exclusivo; n = número de sujeitos incluídos em cada grupo; p = valor de p GP = Grupo Prematuro; GT = Grupo Termo; n = número de sujeitos incluídos

A adesão ao AME, para os dois grupos, foi diminuindo progressivamente no decorrer dos meses. No 30º dia pós-parto o GP apresentou aderência de 100% das mães, contra 78% do GT (p=0,023). A Figura 2 apresenta esses resultados.

**Figura 2:** Número de mães em AME no 30º, 120º e 180º dia de acompanhamento

## DISCUSSÃO

Apesar da maioria das mães de recém-nascidos prematuros e a termo apresentarem uma expectativa alta acerca da autoeficácia do aleitamento materno, não foi encontrada associação da autoeficácia e a prática do AME no período de 180 dias na amostra estudada. Na análise sobre as características socioeconômicas, o grupo de mães de recém-nascidos prematuros apresentou menor renda familiar quando comparado ao grupo de mães de recém-nascidos a termo.

A renda familiar mensal pode provocar mudanças na saúde materno infantil e influenciar as condições de nascimento, assim como acarretar morbidades neonatais e na infância^(21)^. Blencowe et al^(22)^ afirmam que as taxas de nascimento prematuro são mais altas em países de baixa renda, seguidas por países de média renda; o que pode justificar a diferença apresentada em nosso estudo.

O escore de autoeficácia não se mostrou diferente entre o GT e o GP e não foi um preditor para o AME em nenhum momento da pesquisa. Diferente desse resultado, Brandão e colaboradores, em um estudo prospectivo^(23)^, realizado com mães de recém-nascidos a termo, no qual aplicaram a BSES ainda no período gestacional, verificaram associação entre a classificação da BSES e o AME no 30º dia, ao realizarem um acompanhamento da população até o 180º dia de nascimento^(23)^. Não ter incluído mães de prematuros pode ter contribuído com o sucesso da associação no estudo de Brandão. Wang et al e Chantal et al^(15,24)^, destacam que RNPT exigem maior atenção, uma vez que enfrentam obstáculos fisiológicos e de desenvolvimento neurológico relacionados à sua imaturidade^(15)^. Dependendo da idade gestacional, os RNPT apresentam sucção fraca, dificuldade na coordenação respiratória e na deglutição^(24)^.

Todavia, em nosso estudo, apesar da autoeficácia não predizer a adesão ao AME no período de 180 dias, o GP apresentou maior adesão ao AME ao longo de todas as avaliações. Uma hipótese para justificar esse resultado refere-se ao tempo de internação, variável não controlada no presente estudo. Com o tempo de internação mais prolongado, comparado aos recém-nascidos a termo, as mães de RNPT têm a oportunidade de receber, por mais tempo, o serviço de educação continuada a respeito da amamentação e cuidados com o recém-nascido.

Corroborando com a hipótese anteriormente citada, em relação ao tempo de permanência de RNPT em ambiente hospitalar, um estudo realizado no pós-parto imediato, com grupos de educação e apoio organizados por profissionais de saúde especializados no manejo clínico da amamentação e da lactação, relatou que intervenções educacionais realizadas durante a hospitalização podem auxiliar as mães a aumentar a autoeficácia e manter a amamentação exclusiva^(25)^.

Adicionalmente, o tempo de internação pode ter contribuído para uma comunicação regular entre famílias e serviço de saúde, favorecendo o engajamento e a adesão ao estudo após alta hospitalar^(26)^. Um dado para justificar essa nossa hipótese foi a baixa adesão do GT, grupo de recém-nascidos que habitualmente permanece menor tempo internado, se comparado a recém-nascidos prematuros^(27)^. A adesão às intervenções pelos pais ou responsáveis também é fundamental durante o tratamento do recém-nascido, pois permite a definição de metas individualizadas e otimização de cuidados^(26)^.

Em relação à taxa de AME, no 180º dia, 27% dos lactentes estavam em aleitamento materno exclusivo; resultado inferior ao divulgado pelo Ministério da Saúde em 2009^(1)^, no qual a prevalência do AME em menores de 6 meses foi de 41% no conjunto das capitais brasileiras e 40% na cidade de Natal. Outro estudo^(28)^, realizado em uma maternidade amiga da criança, com 261 mães, verificou que a taxa de aleitamento materno exclusivo no 6º mês de vida foi de 5,7%, resultado inferior ao encontrado neste estudo.

De fato, o desmame precoce é um fenômeno complexo e diferentes pesquisas tentam compreender qual a melhor ferramenta para predizer esse risco. Cortelo et al^(29)^, em seu estudo, utilizou o senso de coerência de Antonovsky para explicar este fenômeno, e os resultados apontaram que mães com maior senso de coerência possuem quase duas vezes mais chance de manter o aleitamento por mais tempo. Outro estudo, com crianças menores de 1 ano, relacionou a Escala de Depressão Pós-Parto de Edimburgo com a amamentação nas últimas 24 horas que antecederam a aplicação da escala, o resultado encontrado foi uma maior chance de ausência do aleitamento materno exclusivo entre as mães com sintomas de depressão pós-parto^(30)^.

É conhecido que a prática do AME pode ser influenciada por fatores biológicos^(2)^, psicológicos^(3)^, sociais^(4)^ e culturais^(5)^. E, apesar de investigarmos a prática do AME em um período longo, isto é, por 180 dias, não conseguimos rastrear todos os fatores de risco que poderiam interferir nesse resultado. Um exemplo sobre este aspecto foi a queda brusca de adesão ao AME a partir do 120º dia do GT. Atribuímos esta queda ao fato de muitas mães retornarem aos seus trabalhos após 120 dias de licença, mas não conseguimos investigar esse desfecho. Além disso, algumas variáveis que podem interferir na duração do aleitamento, como o trabalho fora de casa e o uso de pacificadores, não foram analisadas.

Entendemos essa dificuldade de análise de algumas variáveis, que podem ser consideradas importantes, como uma limitação do nosso estudo. Conduzir um estudo longitudinal, com mães de recém-nascidos, no nordeste brasileiro, é uma tarefa árdua e prezamos em manter uma entrevista curta e de fácil execução, para conseguirmos tempo para empoderarmos as mães sobre a importância de manter o AME.

Ter acompanhado as puérperas até o 180º dia de vida do RN foi positivo, visto que poucos estudos conseguem manter um contato tão longínquo nesse tipo de amostra. Entretanto, o primeiro momento escolhido para aplicação da escala BSES pode não ter sido o mais confiável para o relato da autoeficácia da amamentação em mães, dado que foi realizado no pós-parto imediato (entre as primeiras 24 - 48 horas após o parto) e sabe-se que a amamentação nas primeiras horas de vida é desafiadora, confusa e desconhecida tanto para a mãe (principalmente as mães primíparas) quanto para o bebê.

Dessa forma, sugere-se a realização de mais estudos, com a avaliação feita em momentos que não sejam o pós-parto imediato, para confirmar os achados. Uma outra limitação encontrada está no fato da amostra ter sido realizada em uma única maternidade. Assim, mais estudos sob essa mesma perspectiva devem ser conduzidos, a fim de confirmar os dados evidenciados nesta população.

## CONCLUSÃO

Na amostra estudada, a BSES não constituiu um fator preditivo para o desmame precoce tanto em mães de recém-nascidos prematuros, quanto em mães de recém nascidos a termo. O grupo de sujeitos prematuros apresentou maior adesão ao aleitamento materno exclusivo no 30º dia pós parto, além de menor renda familiar. Todavia, novos estudos devem aprimorar esse sistema de monitoramento e investigar diferentes fatores que podem se apresentar como barreiras para a manutenção do aleitamento materno exclusivo.
